# APOE and Alzheimer’s Disease: From Lipid Transport to Physiopathology and Therapeutics

**DOI:** 10.3389/fnins.2021.630502

**Published:** 2021-02-17

**Authors:** Mohammed Amir Husain, Benoit Laurent, Mélanie Plourde

**Affiliations:** ^1^Centre de Recherche Sur le Vieillissement, Centre Intégré Universitaire de Santé et Services Sociaux de l’Estrie-Centre Hospitalier Universitaire de Sherbrooke, Sherbrooke, QC, Canada; ^2^Département de Médecine, Faculté de Médecine et des Sciences de la Santé, Université de Sherbrooke, Sherbrooke, QC, Canada; ^3^Département de Biochimie et Génomique Fonctionnelle, Faculté de Médecine et des Sciences de la Santé, Université de Sherbrooke, Sherbrooke, QC, Canada

**Keywords:** Alzheimer’s disease, APOE, APOE receptors, lipidation, amyloid β, therapeutics

## Abstract

Alzheimer’s disease (AD) is a devastating neurodegenerative disorder characterized by extracellular amyloid β (Aβ) and intraneuronal tau protein aggregations. One risk factor for developing AD is the *APOE* gene coding for the apolipoprotein E protein (apoE). Humans have three versions of *APOE* gene: ε2, ε3, and ε4 allele. Carrying the ε4 allele is an AD risk factor while carrying the ε2 allele is protective. ApoE is a component of lipoprotein particles in the plasma at the periphery, as well as in the cerebrospinal fluid (CSF) and in the interstitial fluid (ISF) of brain parenchyma in the central nervous system (CNS). ApoE is a major lipid transporter that plays a pivotal role in the development, maintenance, and repair of the CNS, and that regulates multiple important signaling pathways. This review will focus on the critical role of apoE in AD pathogenesis and some of the currently apoE-based therapeutics developed in the treatment of AD.

## Overview

Polymorphism in the apolipoprotein E (APOE) gene is a major risk for developing late onset Alzheimer disease (LOAD), whose symptoms are more frequently appearing after the age of 65 years (Yamazaki et al., 2019). The ε4 allele of *APOE* gene is the strongest risk factor for LOAD (Yamazaki et al., 2019). The differences in the structure of apoE isoforms influence their ability to bind lipids, receptors, and amyloid-β (Aβ), which aggregates in plaques within the brain. Human and animal studies clearly indicate that apoE isoforms differentially regulate neuroinflammation, tau hyperphosphorylation, Aβ aggregation and clearance (Tachibana et al., 2019; Kloske and Wilcock, 2020; Vasilevskaya et al., 2020). ApoE regulates lipid homeostasis by mediating lipid transport from one tissue or cell type to another (Holtzman et al., 2012; Chernick et al., 2018; Zhao et al., 2018b). Since lipids such as cholesterol and triglycerides are insoluble in water, they must be carried in the circulation by hydrophile-lipophile particles named lipoproteins. These lipoproteins play a major role in the absorption and transport of dietary lipids between the small intestine, liver and peripheral tissues to the brain where they are essential. In the periphery, it is established how lipids travel in the blood using the different types of lipoproteins (Holtzman et al., 2012; Chernick et al., 2018; Zhao et al., 2018b), whereas within the CNS, lipoproteins are often designated as high-density lipoproteins (HDL)-like, yet their size, shape, and distribution remain unclear. ApoE, present in the CNS and the periphery, represents a critical link between these two compartments and could influence Alzheimer’s disease (AD) pathogenesis by disrupting the blood–brain barrier (BBB) integrity from both sides (Chernick et al., 2019). In this review, the possible mechanisms by which apoE exerts its modulatory effect on AD physiopathology are discussed and new therapeutic perspectives targeting apoE for AD treatment are also described.

## Human APOE Gene

### Gene Polymorphism

**FIGURE 1:**
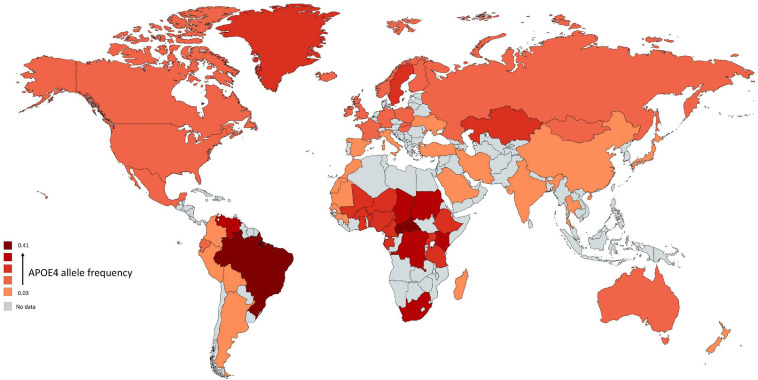
Global distribution of APOE4 allele in *Homo sapiens*. Frequency of ε4 is low (light red regions) and high (dark red regions). The gray color indicates that there are no data available for this country. The geographical map of APOE4 frequency was generated using the package, rworldmapR. State estimates were generated by averaging the frequency of APOE4 allele across the various studies conducted in a specific country.

All species have one version of *APOE* gene while humans have three versions: APOE-ε2 (APOE2), APOE-ε3 (APOE3), and APOE-ε4 (APOE4) allele (McIntosh et al., 2012). The human *APOE* gene comprises of several single-nucleotide polymorphisms (SNPs) distributed across the gene (Nickerson et al., 2000). The most common SNPs are categorized as rs429358 (C > T) and rs7412 (C > T) that lead respectively in amino acid change at position 112 and 158 within the apoE protein (Belloy et al., 2019). The haplotype combination at the two SNPs results in three apoE protein isoforms: apoE2 (Cys112, Cys158), apoE3 (Cys112, Arg158), and apoE4 (Arg112, Arg158). In non-human mammals, APOE genotype is (Thr61/Arg112/Arg158) while all human APOE alleles have an Arginine in position 61 (Arg61). Combinations of these specific amino acids modify the protein structure and functions. The world-wide frequency of human APOE alleles varies considerably (Figure 1). APOE3 is the most common among all human populations and its frequency ranges from 85% (Asia) to 69% (Africa) (Singh et al., 2006). APOE4 allele frequency varies considerably in native populations of Central Africa (40%), Oceania (37%), and Australia (26%) (Corbo and Scacchi, 1999). The distribution across Europe and Asia follows an apparent gradient from north to south, with low APOE4 allele frequencies in the Mediterranean or South China and higher frequencies in northern regions (up to 25%) (Egert et al., 2012). APOE2 is the least common allele with a global prevalence of 7.3% and is absent in many indigenous people without any clear regional pattern (Corbo and Scacchi, 1999; Singh et al., 2006).

### APOE Allele and Risk of Diseases

The different alleles of *APOE* confer differential risks of developing pathologies (Wu et al., 2018). A meta-analysis of clinical and autopsy-based studies on five ethnic groups (Caucasian, African American, Hispanic, and Japanese) revealed that among Caucasian subjects, the risk of developing AD was increased in individuals with one APOE4 copy compared to individuals homozygote for APOE3 (Farrer et al., 1997). Compared to non-carriers of APOE4, the increased risk of AD is 3–4 fold in heterozygotes and about 9–15 fold in APOE4 homozygotes (Farrer et al., 1997; Neu et al., 2017). The APOE4-AD correlation was weaker among African–Americans and Hispanics, and greater in Japanese people compared to Caucasian cases (Table 1). The risk of AD was however decreased in people carrying APOE2 compared to those carrying APOE3 (Farrer et al., 1997). According to a population-based cohort study, lifetime risk of mild cognitive impairment (MCI) or dementia is 30–35% for APOE4 homozygote individuals, 20–25% for APOE4 heterozygote individuals (ε3/ε4 and ε2/ε4), and 10–15% for non APOE4 individuals (ε3/ε3, ε3/ε2, and ε2/ε2) (Qian et al., 2017). With respect to other APOE genotypes, familial type III hyperlipoproteinemia is associated to those homozygous for APOE2 (Yang et al., 2007; McIntosh et al., 2012). Altogether, these APOE-related risks for diseases point toward differences in the structure and function of the proteins involved in lipid metabolism.

**TABLE 1:** APOE genotypes, allele frequency distribution, and odds ratio for developing AD, stratified by AD patient cases and controls on five ethnic groups (Farrer et al., 1997).

		APOE genotype {(frequency (%) /						APOE allele		
		AD Odds ratio (95 % confidence interval)}						frequency (%)		
Ethnic groups	No	E2/E2	E2/E3	E2/E4	E3/E3	E3/E4	E4/E4	E2	E3	E4
CAUCASIAN CASE PATIENTS	5107	0.2/0.9	4.8/0.6	2.6/1.2	36.4/1.0	41.1/2.7	14.8/12.5	3.9	59.4	36.7
Controls	6262	0.8	12.7	2.6	60.9	21.3	1.8	8.4	77.9	13.7
AFRICAN AMERICAN	235	1.7/2.4	9.8/0.6	2.1/1.8	36.2/1.0	37.9/1.1	12.3/5.7	7.7	59.1	32.2
CASE PATIENTS										
Controls	240	0.8	12.9	2.1	50.4	31.8	2.1	8.3	72.7	19.0
HISPANIC	261	0.4/2.6	9.6/0.6	2.3/3.2	54.4/1.0	30.7/2.2	2.7/2.2	6.3	74.5	19.2
CASE PATIENTS										
Controls	267	0.4	12.0	0.8	67.4	17.6	1.9	6.7	82.3	11.0
JAPANESE	336	0.3/1.1	3.9/0.9	0.9/2.4	49.1/1.0	36.9/5.6	8.9/33.1	2.7	69.5	27.8
CASE PATIENTS										
Controls	1977	0.4	6.9	0.8	75.7	15.5	0.8	4.2	86.9	8.9

### APOE2, Guardian Angel Against AD?

A study on 38,537 people from six population-based cohorts showed a survival benefit for APOE2 carriers (Wolters et al., 2019). They identified 239 APOE2 homozygotes, who have lived the longest lives. Another group reported that compared with the ε3/ε3 genotype, individuals with the ε2/ε2 genotype have larger gray-matter volume in brain areas subjected to AD (i.e., hippocampi, medial temporal inferior temporal, cortex, precuneus, superior parietal regions, and temporal pole) and in areas related to cognitive resilience during aging (i.e., anterior cingulate and medial prefrontal areas) (Arenaza-Urquijo et al., 2019). APOE2 homozygotes have a 66% reduction in AD risk compared to ε2/ε3 carriers, an 87% reduction in AD risk compared to APOE3 homozygotes, and a 99.6% reduction in AD risk when compared to APOE4 homozygotes (Reiman et al., 2020). These recent studies on APOE2-related genotypes might stimulate research interest for characterizing the molecular advantages of apoE2 protein over the other isoforms.

## APOE Protein

### Structure

**FIGURE 2:**
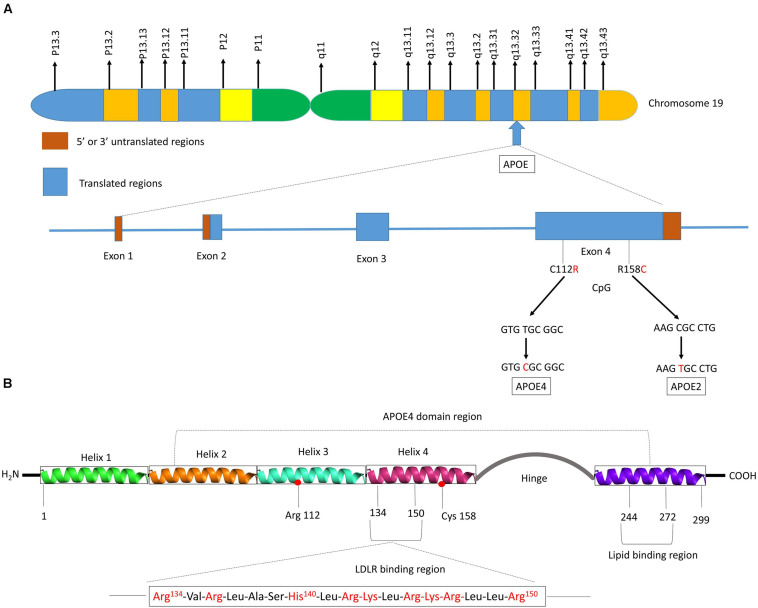
Schematic illustration of structural and functional regions of apoE protein. **(A)** Location and structure of the *APOE* gene on chromosome 19 at position q13.32. The *APOE* gene has 4 exons, respectively consisting of 44, 66, 193, and 860 nucleotides, with exon 4 coding for over 80% of the protein. Exon 1 contains the 5′ untranslated region (5′UTR). The wild-type sequence is apoE3 (Cys112, Arg158). Point mutations in the exon 4 generate two single nucleotide polymorphisms (SNPs): a T → C point mutation produces apoE4 (C112R), while C → T mutation gives apoE2 (R158C). **(B)** Diagram of human apoE structural domains. The N-terminal region contains the receptor-binding site (residues 134–150) and four helices (1–4), the C-terminal region contains the lipid-binding region (residues 244–272) and the two domains are joined by a hinge region. The N-terminal region contains the two polymorphic positions (112 and 158) that discriminate the three apoE isoforms. The lower part of the figure shows the α-helical segment (residues 134–150) that recognizes the LDL receptor. This segment is rich in positively charged arginine and lysine residues.

Human *APOE* gene is located on the chromosome 19 at position q13.32 (Figure 2A) and codes for a 299 amino acid protein (∼36 kDa) whose primary function in the brain is to transport cholesterol. ApoE contains three main regions: a N terminal region containing the receptor-binding site and four helices, a C-terminal region containing the lipid-binding site and three helices, and an intervening flexible hinge region that links the N- and C-terminal regions (Figure 2B) (Liu et al., 2013; Flowers and Rebeck, 2020). ApoE isoforms differ by a unique amino acid combination at position 112 and 158: apoE2 (Cys112, Cys158), apoE3 (Cys112, Arg158), and apoE4 (Arg112, Arg158) (Yu et al., 2014). The genotype-related change of one or two amino acids within the apoE protein modifies its structure. For instance, Cys-158 in apoE2 removes a salt-bridge between Arg158 and Asp154, reduces the positive potential, and consequently changes the receptor binding region (Wilson et al., 1991). Arg-112 changes the lipid binding region of apoE4 and shifts the lipid binding preference from HDL to very-low-density lipoproteins (VLDL) (Mahley and Rall, 2000). The existing interaction between the amino acid 61 and 112 influences the lipoprotein binding; it is the main reason explaining the high affinity of apoE4 to VLDL while apoE2 and apoE3 bind to HDL (McIntosh et al., 2012). Even though apoE of the non-human mammals such as chimpanzee is similar to the human ε4 allele, the presence of a threonine at position 61 (Thr61) makes it work more like the human ε3 allele (McIntosh et al., 2012).

### Tissue Expression

The human *APOE* gene is expressed in several organs and in various cell types. Ninety percent of the circulating apoE is produced by the liver (Yu et al., 2014) and to a lesser extent by the adrenal gland and macrophages (Kockx et al., 2008). Other cells capable of synthesizing apoE include astrocytes, macrophages, and endocrine cells such as ovarian and adrenal cells (Huang et al., 2015). In the CNS, apoE secretion is sustained by astrocytes, oligodendrocytes, pericytes, choroid plexus and neurons (Kang et al., 2018; Flowers and Rebeck, 2020). ApoE lipoproteins produced by the choroid plexus are directly secreted into the cerebrospinal fluid (CSF) (Achariyar et al., 2016). ApoE is also synthesized by glial cells and associates with lipids to form lipid-transport particles in the CSF (Koch et al., 2001).

The expression level of apoE varies by genotype. For instance, individuals carrying APOE2 have higher concentration of apoE proteins in the CSF whereas APOE4 carriers have lower levels (Castellano et al., 2011; Cruchaga et al., 2012). The same observation was made in a mouse model whose *APOE* gene was replaced by the human APOE2 (hAPOE2) or hAPOE4 (Ulrich et al., 2013). ApoE levels in the CNS of AD patients are inconsistent with studies reporting either higher (Baker-Nigh et al., 2016), lower (Cruchaga et al., 2012; Talwar et al., 2016) or unchanged level of apoE (Martínez-Morillo et al., 2014) compared to healthy individuals. However, the expression levels of APOE mRNA in post-mortem AD brain tissues are elevated compared to controls (Gottschalk et al., 2016), emphasizing the difficulty to correlate APOE mRNA and protein levels.

## APOE Receptors

### Affinity for apoE Isoforms and Their Tissue Expression

ApoE is a ligand for cell surface lipoprotein receptors belonging to the low-density lipoprotein receptor (LDLR) family (Holtzman et al., 2012). The LDLR family consists of eight receptors i.e., LDLR, very-low-density lipoprotein receptor (VLDLR), apolipoprotein E receptor 2 (apoER2 or LRP8), LRP4, LDLR-related receptor 1 (LRP1), LRP1b, megalin (LRP2), and LR11/SorLA (Lane-Donovan and Herz, 2017). All LDLR family members share structural properties that allow them to interact with apoE isoforms but with distinct affinities. ApoE3 and apoE4 isoforms bind with high affinity to the LDLR and LRP1 (Ruiz et al., 2005; Zhao et al., 2018b) whereas apoE2 binding to LDLR is 50 times weaker than that of apoE3 or apoE4 (Hatters et al., 2006a). VLDL receptor recognizes all apoE isoforms with equal affinity (Ruiz et al., 2005). Co-immunoprecipitation assays revealed differences in the formation of the apoE-LR11 complex for each isoform (apoE4 > apoE3 > apoE2, apoE4 binding the most), however the lipidation and oxidation status of apoE was not addressed in this study (Yajima et al., 2015). It remains unclear whether apoE differentially activates apoE receptors.

These receptors are expressed by many tissues. LDLR, is expressed by neurons and hepatocytes (Goldstein and Brown, 2015). LRP1 is ubiquitously expressed, but more abundantly in vascular smooth muscle cells (SMCs), hepatocytes, and neurons (Etique et al., 2013). LRP1 is present on plasma membrane of various cells including microglia, astrocytes, and neurons. LRP1b is primarily expressed in the brain, and a differentially spliced form is present in the adrenal gland and in the testis (Marschang et al., 2004). VLDLR is expressed by neurons and in tissues throughout the body while apoER2 is restricted to the brain, testis, and placenta (Dlugosz and Nimpf, 2018). Megalin is highly expressed in the proximal tubule of the kidney (Christensen et al., 1992) and in many other absorptive epithelia, e.g., lung, retina, yolk sac, inner ear, and brain (Kounnas et al., 1994). LR11 is expressed in neurons of the central and peripheral nervous system (Kim et al., 2009).

### ApoE Receptors and AD

The role in AD pathogenesis for each member of the LDLR family remains unclear. Overexpression of LDLR in the brain could lower apoE levels, increase the clearance of Aβ which is one of the neurological hallmarks of AD, and decrease the deposition of Aβ (Kim et al., 2009). LRP1 could affect AD pathogenesis by controlling amyloid precursor protein (APP) processing and Aβ catabolism (Cam and Bu, 2006). Alterations in APP cellular trafficking and localization directly impact its processing to Aβ, and disrupting the interaction between LRP and APP could decrease Aβ production which in turn affects development of AD (Cam and Bu, 2006). LRP1b binds to APP and decreases the processing of APP to Aβ (Cam et al., 2004). Hence, the expression of LRP1b might also be involved in protecting against the pathogenesis of AD by decreasing the generation of Aβ proteins within the CNS (Cam et al., 2004). ApoE isoforms also affect the endocytosis of receptors. For instance, carrying the APOE4 allele significantly impairs the recycling of apoER2 and VLDLR compared to APOE3 and APOE2 (Chen et al., 2010). Since apoER2 interacts with APP and affects APP processing, lower recycling might increase output of Aβ (He et al., 2007). Unlike other members of the LDLR family, SorLA expression does not affect APP endocytosis, but rather mediates APP intracellular transport processes (Kim et al., 2009). Megalin was thought to clear Aβ from the brain at the choroid plexus across the blood-CSF barrier (Spuch et al., 2015). It has been shown that binding of megalin to Aβ is decreased in the CSF of AD patients, suggesting that decreased Aβ sequestration in the CSF could be associated with defective Aβ clearance and increased brain Aβ levels (Spuch et al., 2015).

## Metabolism and APOE Isoforms

### Lipidation of apoE Isoforms

To perform its important functions (i.e., cholesterol transport, immune modulation, synapse regeneration, and clearance/degradation of Aβ), apoE must be secreted and properly lipidated (Kanekiyo et al., 2014; Hu et al., 2015). ApoE lipidation is facilitated by the cholesterol-efflux protein ATP-binding cassette A1 (ABCA1). ABCA1 is present in a wide variety of body cells, including the brain’s astrocytes, neurons, BBB and in the choroid plexus (Flowers and Rebeck, 2020). ABCA1 is essential for the proper lipidation of apoE and absence of ABCA1 in knockout mice leads to a decrease in the overall apoE level in the CNS (Flowers and Rebeck, 2020). ApoE isoforms differ in their lipid binding and lipoprotein preferences. The C-terminal domain of apoE (273–299) is critical for the lipoprotein binding and therefore determines apoE isoform lipidation specificity and efficiency (Hu et al., 2015). Contrary to apoE2 and apoE3, apoE4 is poorly lipidated (Kanekiyo et al., 2014). ApoE3 and apoE2 preferentially bind to small phospholipid-rich HDL while apoE4 strongly binds to large triglyceride-rich VLDL (Nguyen et al., 2010). Reduced binding affinity of apoE4 for HDL results in a greater proportion of unlipidated apoE, hence forming aggregates (Hatters et al., 2006a). ApoE4 large aggregates are more toxic for neurons than apoE2 and apoE3 aggregates (Hatters et al., 2006b). Biophysical studies have shown that lipid-free apoE appears to aggregate *in vitro* in an isoform-dependent manner (apoE4 > apoE3 > apoE2), and lipidation of apoE impedes aggregates formation (Hubin et al., 2019). Unlipidated apoE monomers form multimers such as dimers and tetramers, and apoE can additionally aggregate to form fibrils (Flowers and Rebeck, 2020). Free cholesterol, phospholipids, and triglycerides are the main lipids present in apoE-containing CNS particles (Peng et al., 2003). ApoE4-containing particles have less cholesterol than those containing ApoE3 (Zhao N. et al., 2017). APOE genotypes also modify lipidation states in the periphery. In the homozygous and heterozygous (ε3/ε4) genotypes, APOE4 is frequently associated with increased LDL-cholesterol levels in plasma while in homozygous and heterozygous (ε2/ε3) genotypes, APOE2 is correlated with mild or low levels (Villeneuve et al., 2015). APOE2 genotype correlates with higher triglyceride levels compared to APOE3 and APOE4 (Zhao N. et al., 2017). The amino acid substitution in apoE2 impairs its binding with LDLR and impairs clearance of triglyceride-rich lipoprotein remnant particles, leading to the onset of type III hyperlipoproteinaemia (Phillips, 2014).

### Lipid Transport Within the CNS

ApoE mediates delivery of cholesterol and other lipids to neurons and glial cells. Cholesterol cannot not cross the BBB and the choroid plexus Blood-CSF Barrier (BCSFB), but it is converted into 24S-hydroxycholesterol by the enzyme 24-hydroxylase cholesterol, specifically located in neurons and 24S-hydroxycholesterol can bind to apoE and easily cross the BBB (Russell et al., 2009). It was suggested that apoE4 is involved in AD pathogenesis by mechanisms linked to the metabolism of brain lipids (Hauser et al., 2011). In cultured neurons, apoE4 was less effective than apoE2 and apoE3 to transport brain cholesterol (Rapp et al., 2006). Because cholesterol and phospholipids transport relies on apoE isoforms (apoE2 > apoE3 > apoE4) (Hara et al., 2003), the lower efflux of cholesterol and phospholipids by apoE4 might be involved in the increased risk of LOAD in APOE4 carriers. Two recent studies on human iPSCs-derived astrocytes have shown higher cholesterol accumulation inside apoE4-expressing astrocytes than inside apoE3 astrocytes (Lin et al., 2018; Tcw et al., 2019), supporting that the transport of cholesterol out of the astrocytes might be deficient in APOE4 carriers. A more recent study using iPSC-derived astrocytes showed that apoE4 is less lipidated than apoE3, potentially impacting apoE4 neurotrophic role (Zhao J. et al., 2017). In the CSF of middle-aged adults (average age 54.5 years) with no dementia, apoE particles were smaller in both ε3/ε4 and ε4/ε4 individuals than in ε3/ε3 individuals, but larger in ε2/ε3 individuals (Heinsinger et al., 2016; Nelson and Sen, 2019). The cholesterol efflux ability of individuals homozygous for APOE4 is reduced in CSF (Yassine et al., 2016). The larger particle size in APOE4 homozygote AD patients may inhibit particle binding or endocytosis, thus depriving neurons of enough cholesterol for repair (Yassine et al., 2016). In the CNS, apoE lipid transport capability could also be influenced by the quantity of apoE (Rebeck, 2017). In mice, the amount of apoE in brain parenchyma (Riddell et al., 2008) and CSF (Ulrich et al., 2013) has an isoform-dependent gradient (apoE2 > apoE3 > apoE4). In humans, there was however no isoform-dependent variations in levels of apoE in the CSF in young control subjects (average 34.5 years) (Baker-Nigh et al., 2016), cognitively healthy subjects (average 61 years) and AD patients (average 78 years) (Martínez-Morillo et al., 2014). In a cohort of Aβ-positive cognitively healthy individuals as well as with MCI, levels of apoE in CSF were significantly lower in APOE4 carriers relative to non-carriers (Baker-Nigh et al., 2016). This indicates that extensive lipid homeostasis studies are required to unravel a more comprehensive mechanism.

## APOE Isoforms and AD Physiopathology

**FIGURE 3:**
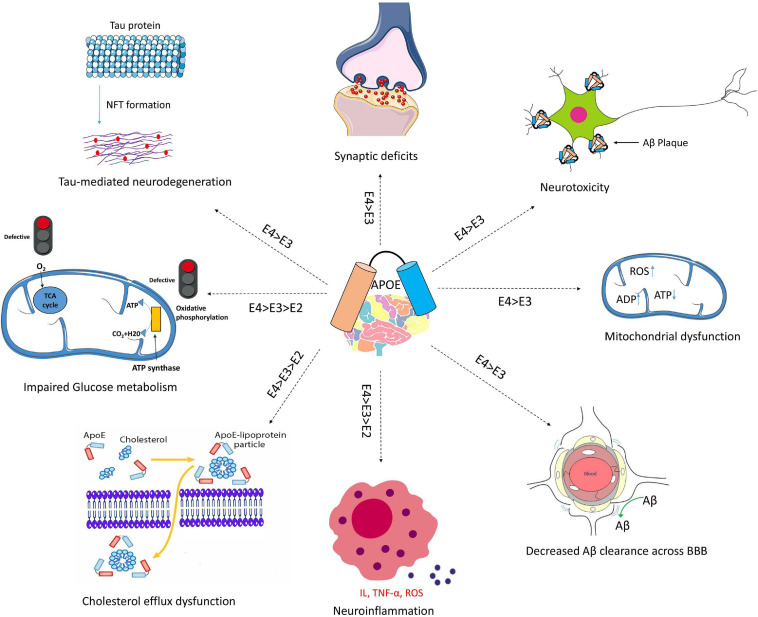
Schematic overview of Aβ-independent roles for apoE in Alzheimer’s disease pathology. The isoform-dependent effects of *APOE* are indicated. Abbreviations: apoE, apolipoprotein E; NFT, neurofibrillary tangle; BBB, blood brain barrier; IL, interleukin; TNF-α, Tumor necrosis factor-α; ROS, reactive oxygen species.

**FIGURE 4:**
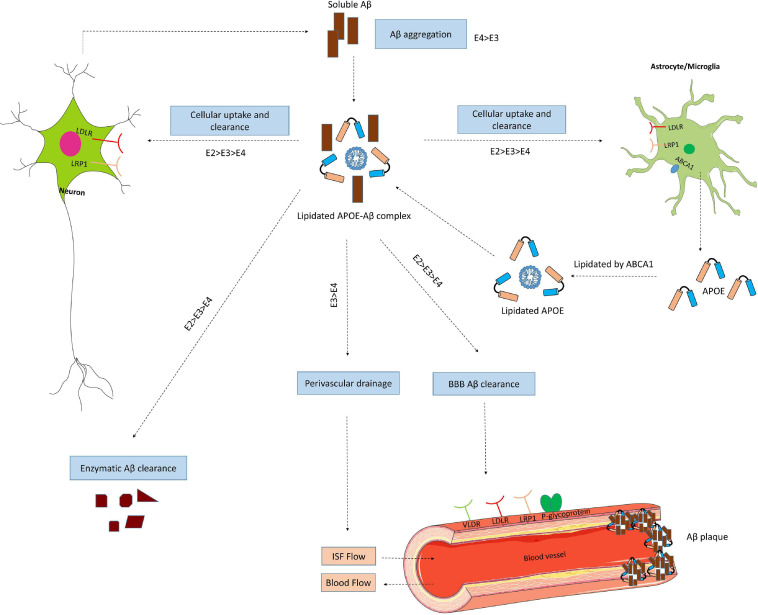
ApoE isoform-dependent effects on Aβ metabolism and clearance. Aβ is mainly produced by neurons via proteolytic cleavage of APP. In the brain, apoE is primarily produced by astrocytes and microglia, and is then lipidated by ABCA1 to form lipoprotein particles. ApoE accelerates isoform-dependent aggregation and deposition of Aβ, but also promotes the cellular uptake and clearance of Aβ by astrocytes or microglia by endocytosis of the lipidated APOE-Aβ complex. This endocytosis is mediated by various receptors, including LDLR and LRP1. ApoE facilitates isoform-dependent extracellular proteolytic degradation of Aβ. At the BBB, soluble Aβ is mostly transported via LRP1 and P-glycoprotein from the interstitial fluid (ISF) into the bloodstream. ApoE also mediates perivascular drainage of Aβ. Insufficient Aβ clearance can cause Aβ aggregation in brain parenchyma and can contribute to the formation of Aβ oligomers and amyloid plaques.

APOE genotypes can affect many cellular functions such as synaptic integrity, lipid transport, glucose metabolism, Aβ clearance, BBB integrity or mitochondria regulation (Figure 3). For instance, recent reviews came to the conclusion that apoE4 increases the pro-inflammatory response, which in turn causes the dysfunction of BBB, and leads to cognitive deficits (Marottoli et al., 2017; Teng et al., 2017; Kloske and Wilcock, 2020). ApoE isoforms also affect the primary neuropathological markers of AD: neuroinflammation, Aβ plaques and tau protein aggregations. Studies in humans and transgenic mice showed that brain Aβ levels and amyloid plaque loads are higher in APOE4 carriers than the other genotypes, with the lowest levels in APOE2 carriers (Huang et al., 2017; Safieh et al., 2019; Tachibana et al., 2019). The increase in Aβ plaques in APOE4 carriers may be due to the enhanced ability of apoE4 to bind Aβ but also its inability to completely remove Aβ from the brain (Kloske and Wilcock, 2020). Other mechanisms involve apoE4 in different pathways such as interstitial fluid (ISF) drainage, uptake by microglial phagocytosis (Figure 4), that could contribute to the decrease of Aβ removal (Castellano et al., 2011; Tarasoff-Conway et al., 2015; Ma et al., 2018). Tau protein hyperphosphorylation as well as the formation of tangles differ by APOE genotype. Overexpression of apoE4 in neurons abnormally increases tau phosphorylation while apoE3 overexpression has no effects (Cao et al., 2017; Shi et al., 2017; Wang et al., 2018; Vasilevskaya et al., 2020). ApoE directly inhibits GSK-3β-mediated phosphorylation of tau (Hoe et al., 2006). In the next sections we will discuss synergy between apoE lipidation and sex specificity with LOAD.

### Crosstalk Between apoE Lipidation and AD

Lipidation of apoE and lipid transport within the CNS are currently under investigation to clarify their roles in the development of LOAD. ApoE is unique among apolipoproteins with its minimal intracellular degradation (Yassine and Finch, 2020). Internalized lipids are dissociated from apoE into late endosomal compartments after intracellular absorption of apoE-containing lipoprotein particles, followed by recycling of apoE into early endosomes and its re-secretion within or into HDL particles (Yassine and Finch, 2020). ApoE4 has lower recycling capacity due to its greater affinity for lipid binding. This property reduces the efflux of cholesterol and enriches the cell membrane with cholesterol (Yassine and Finch, 2020). ApoE recycling controls the expression of several cell surface proteins, such as ABCA1 (Rawat et al., 2019), the insulin receptor (IR), or LRP1 (Yassine and Finch, 2020). The reduced apoE4 recycling traps ABCA1 in endosomes, away from the cell surface. Reduced activity of ABCA1 hence contributes to lower efflux of cholesterol to HDL and redistributes cholesterol to cell membranes (Rawat et al., 2019). Greater cell membrane cholesterol enhances TLR4 signaling in macrophages which in turn, activates NFkB and induces inflammatory genes response (Singh et al., 2020; Yassine and Finch, 2020). Lower apoE4 recycling in the brain also traps insulin receptor (IR) away from cell surface in the endosome (Zhao N. et al., 2017), hence modifying its preferences for cellular energy sources. Consequently, it reduces glucose utilization to generate ATP and encourages oxidation of fatty acids (Svennerholm et al., 1997). It is reported that the level of phospholipids (PL) decreases in the brain by 42% between the age of 20 and 100 years old, and that there is an additional loss of 20% in the AD brain (Mesa-Herrera et al., 2019). In humans, our group also showed that beta-oxidation of docosahexaenoic acid (DHA), a polyunsaturated fatty acid that is highly concentrated in brain membranes, is higher in APOE4 carriers than the non-carriers (Chouinard-Watkins et al., 2013). Since the brain largely relies on glucose to fulfill its high energy-demand, the decrease of glucose uptake through the BBB during aging had been identified as a risk factor for developing AD (Fernandez et al., 2019). This is especially true in APOE4 carriers since it was reported that human astrocytes expressing apoE4 have half of the glucose uptake capacity compared to apoE3 ones whereas astrocytes expressing apoE2 have the highest glucose uptake (Williams et al., 2020). Moreover, apoE4 proteins secreted from primary astrocytes are poorly lipidated compared to apoE3. Increasing the activity of ABCA1 could therefore provide a therapeutic approach to promote the recycling of apoE4 from endosomes and restore its function at the membrane level (Yassine and Finch, 2020). A greater distribution of cholesterol at the neuronal plasma membrane increases the expression of BACE1 and APP processing to generate more Aβ (Cui et al., 2011). In astrocytes and microglia, less efflux of cholesterol decreases Aβ degradation which in turn might accentuate its aggregation to form plaques. In astrocytes, LRP1 complexes with apoE and the reduced plasma membrane recycling of LRP1 decreases the ability of astrocytes to degrade Aβ peptides (Prasad and Rao, 2018), providing one mechanism for the increased development of apoE4 associated amyloid plaques. Altogether, there are emerging evidence that in APOE4 carriers, there are metabolic shifts in the energy metabolism that could contribute to LOAD pathogenesis during aging.

One interesting observation in individuals carrying one or two APOE4 copies have usually “normal” brain functions until older ages despite having low lipidation of their apoE4 protein. Therefore, apoE4 lipid transport capabilities to supply neurons and astrocytes are probably decreased in late stages of life in APOE4 carriers. Hence, a young brain might have mechanisms in place to cope with inadequate lipid transport related to apoE4 protein structure (Yassine and Finch, 2020). During aging, there is a potential loss of these alternative mechanisms while there is also a decreased production of cholesterol (Boisvert et al., 2018), and both could lead to neuronal lipid deficits (Fernandez et al., 2019). Identification of the coping mechanisms that are lost during aging would highly benefit the field in moving forward this research area.

### Sex-Specificity in LOAD and for apoE4 Lipidation

The risk of developing LOAD is much higher for women than men (Barnes et al., 2005). Women with a single APOE4 copy have a significant increase risk of AD compared to men carrying two APOE4 copies (Farrer et al., 1997). The risk factor for women with one APOE4 copy is around 4-fold whereas in men with one APOE4 copy, the risk is 1-fold higher only (Payami et al., 1994; Altmann et al., 2014). There are also marked regional variations between men and women in the regulation of fatty acid metabolism. Triglycerides (TG) are distributed differently in the adipose tissue of male and females. Moreover, concentrations of polyunsaturated fatty acids in the adipose tissue are higher in pre-menopause women compared to men (Lohner et al., 2013; Lim et al., 2020). Estrogen levels, higher in women before menopause, play a key role in the transport of lipids, increasing metabolic enzyme expression, and reducing α-linolenic acid (ALA) oxidation, an essential polyunsaturated fatty acid (Palmisano et al., 2018). In post-menopausal women, the decrease in estrogen levels is associated with increased plasma TG levels and lower HDL, both of which increase the risk of cognitive decline (Ancelin et al., 2014; Chew et al., 2020). Higher rate of cognitive decline was observed with increased TG content and lower HDL levels (Ancelin et al., 2014). APOE4 allele increases the risk of abnormal Aβ aggregation in men and women equally, but impacts tau hyperphosphorylation more prominently in women (Altmann et al., 2014). Women APOE4 carriers with MCI had higher CSF tau and tau/Aβ ratios compared to APOE4 males with MCI (Payami et al., 1994). Likewise, women APOE4 carriers with mild LOAD had a greater risk of developing both neurofibrillary tangles and Aβ plaques than APOE4 men with mild LOAD (Corder et al., 2004). However, women and men carrying two APOE4 copies have both a 15-fold higher risk of developing LOAD. The sex-specific differences of APOE4 genotype can also occur at the level of gene regulation as apoE4 functions as a transcription factor in the brain, binding to the promoter regions of genes linked to microtubule disassembly, programmed cell death, synaptic function, and aging (Theendakara et al., 2016). ApoE4-mediated transcriptional activity is sex-specific for genes involved in the response of the immune system, inflammation, oxidative stress, aging and estrogen signaling as different patterns of activation have been observed between female and male ε4-positive brains (Hsu et al., 2019). Collectively, these findings show that there are sex differences in the risk of developing LOAD based on the APOE genotype. Therefore, sex should be considered when investigating the impact of different therapeutic strategies on the modulation of lipid metabolic pathways.

## APOE-Targeted Therapies for LOAD

Carrying the APOE4 allele is associated with higher deposition of Aβ in the brain however it remains to be proved that Aβ is AD-causative (Bu, 2009) since Aβ deposition in human brain without significant cognitive impairment are frequently observed (Aizenstein et al., 2008). Moreover, clinical trials reporting reduction of brain Aβ accumulation do not necessarily show improvement of cognition supporting that there is no direct link between Aβ plaque reduction and improved cognition (van Veluw et al., 2017). As the disease is complex and multifactorial, it is likely that the current developed drugs target the wrong pathological substrates, or that a multi-target drug approach could be required. We describe here apoE-targeted therapies tested in animals and those in the early phases of clinical trials. The current therapeutic strategies targeting apoE to treat LOAD include: (1) targeting apoE structural properties and interaction with Aβ, (2) modulating apoE level and lipidation, (3) targeting APOE receptors, and (4) apoE gene therapies.

### Targeting apoE Structural Properties and Interaction With Aβ

Blocking apoE-Aβ interaction with peptide mimics might be advantageous since the peptide can be very selective due to its precise target (Cesa et al., 2015). Aβ12-28P, a peptide corresponding to residues 12–28, reduces Aβ deposition and insoluble tau accumulation in the brain of mice (Liu et al., 2014), Treatment with Aβ12-28P reduces accumulation of Aβ in brain, co-deposition of apoE within Aβ plaques and neuritic degeneration in APOE2-TR and APOE4-TR Aβ mouse models (Pankiewicz et al., 2014). 6KApoEp is a peptide that inhibits apoE binding to the N-terminus of APP (Sawmiller et al., 2019). Notably, in 5XFAD mouse model, 6KApoEp injection reduces both Aβ and tau pathologies concomitantly with improved memory and hippocampal-dependent learning (Sawmiller et al., 2019). These findings indicate that blockers of apoE-Aβ interaction may potentially be used to reduce the therapeutic burden of Aβ and tau in the CNS.

It is possible to control the lipidation and secretion of apoE using apoE mimic peptides (Osei-Hwedieh et al., 2011). Mimic peptides correspond to the LDL receptor binding domain (130–150 residues) of the apoE protein. These peptides are designed to promote cholesterol trafficking but also alter APP trafficking and processing, and anti-inflammatory signaling within macrophages (Yao et al., 2012). ApoE mimetic peptides, such as 4F, COG112, COG133, and COG1410, increase apoE lipidation and apoE secretion, decrease Aβ levels and tau hyperphosphorylation, inhibit neurodegeneration and neuroinflammation, and improve cognitive functions (Chernick et al., 2018). A recent study in E4FAD mice showed that transient treatment with CN-105 decreases Aβ pathology and rescued memory deficits (Krishnamurthy et al., 2020). Mimic peptide CN-105 has completed Phase I clinical trial (NCT03168581 and NCT03802396) in patients with intracerebral hemorrhage (Guptill et al., 2017). This peptide is derived from the receptor binding region of apoE-α helix and decreases neuronal injury and neuroinflammation in acute brain injury mouse models (Laskowitz et al., 2017; Liu et al., 2018). However, in the context of human apoE isoforms, the effects of these peptides on Aβ deposition and other LOAD-related pathologies have not been thoroughly identified. Therefore, this strategy seem to have gather some success although it still requires to be improved, tested in specific population and to prove its efficacy on cognition to become a therapy.

Another therapeutic strategy is to disrupt apoE4 interaction domain with small molecules, modify apoE4 overall structure and therefore modulate its adverse effects in LOAD pathogenesis (Brodbeck et al., 2011). At least three regions (15–30, 116–123, and 271–279 peptides) vary between apoE isoforms, and targeting these regions with small molecules to switch apoE4 toward an apoE2 and apoE3-like structure appears to be a more direct approach to modulate apoE4 pathobiology (Frieden and Garai, 2012). Small molecules such as PH-002, GIND-25, and CB9032258 (a phthalazinone derivative) target the interaction domain and thus modify the detrimental effects of apoE4 in human neurons (Lin et al., 2018; Wang et al., 2018). This approach is currently being further developed to verify whether it has therapeutic benefits *in vivo*.

### Modulating apoE Level and Its Lipidation

Instead of converting apoE4 structure, another strategy is to use anti-apoE4 antibodies to neutralize the toxic effects of apoE4 (such as Aβ plaques), like the anti-Aβ-based immunotherapies. Such antibodies can cross the BBB even though only a small amount will penetrate the brain effectively (Kim et al., 2012). Anti-apoE antibodies in mouse models were shown to effectively prevent deposition of Aβ when added with pre-existing Aβ deposits (Kim et al., 2012). In a subsequent study, administration of anti-apoE antibodies directly into the brain prevented deposition of new Aβ plaques as well as pre-existing plaques that were cleared. It is very exciting that these anti-apoE antibodies can interfere with the direct binding of apoE to Aβ deposits, as this may act synergistically with anti-Aβ immunotherapy in APOE4 patients to attain a higher degree of Aβ reduction (Liao et al., 2014). A recent study has shown that the antibody HAE-4, which preferentially binds the non-lipidated forms of apoE4/apoE3, is highly effective in reducing the deposition of Aβ in an APP/APOE4 mouse model when delivered directly into the brain by intracerebroventricular injection (Liao et al., 2018). Further testing of this approach are underway to verify the off-target effect of these antibodies that could also detrimentally interfere with the physiological functions of apoE.

Instead of using an antibody to neutralize apoE4, another strategy is to use antisense oligonucleotides (ASO) which can target APOE4 mRNA and decrease its expression (Schoch and Miller, 2017). Reduction of apoE expression by ASO significantly decreased Aβ pathology during the early stages of plaque formation in APP/PS1-21 mice homozygous for APOE4 or APOE3 (Huynh et al., 2017). ASO therapies targeting APOE receptors have also been tested in AD mouse models and treatment of AD mouse with an anti-APOER2 oligonucleotide resulted in increased synaptic function and improved learning and memory functions (Hinrich et al., 2016). There are only few ASO-mediated therapies in clinical trials for AD, the most prominent one being the anti-tau ASO in phase1/2 trial (BIIB080 from Ionis/Biogen/Washington University) (DeVos et al., 2017) but results are, to our knowledge, not yet published.

ApoE4 is hypolipidated suggesting that the pathological effects depend on how much apoE4 is lipidated. Since ABCA1 plays an important role in apoE lipidation, some groups worked on increasing ABCA1 activity to improve apoE4 lipidation. Bexarotene and 9-cis retinoic acid are drugs able to regulate ABCA1 expression (Boehm-Cagan et al., 2016; Tachibana et al., 2016). In an Aβ mouse model expressing human apoE4 and apoE3, treatment with bexarotene and 9-cis retinoic acid increased ABCA1 levels in both mice groups and reversed Aβ and hyperphosphorylated tau accumulation in hippocampal neurons, as well as cognitive deficits (Tachibana et al., 2016). Intraperitoneal injection of CS-6253 injection, an ABCA1 agonist peptide, increased apoE4 lipidation, decreased Aβ accumulation and tau hyperphosphorylation as well as reduced cognitive deficits in APOE4-TR mice (Boehm-Cagan et al., 2016). Taken together, these studies show that modulating apoE4 lipidation by increasing ABCA1 expression reduced Aβ accumulation and thereby cognitive deficits.

### Targeting apoE Receptors

Considering that Aβ clearance in the brain is partially mediated by apoE receptors, especially LRP1, LDLR, and APOER2, increasing the expression of these receptors is a possible therapeutic strategy for reducing Aβ pathology. Fluvastatin, a hydroxymethylglutaryl-CoA reductase inhibitor, decreases Aβ deposition and enhances Aβ clearance in cultured brain microvessel endothelial cells, possibly by increasing LRP1 expression (Qosa et al., 2012). Another study found that increased Aβ clearance in brain endothelial cells and isolated mice brain microvessels treated with rifampicin or caffeine (Shinohara et al., 2010). In Aβ mouse models, conditional LRP1 knockout in neurons (Kanekiyo et al., 2013), astrocytes (Liu et al., 2017), and vascular SMCs (Kanekiyo et al., 2012) resulted in increased deposition of Aβ. In APOE4-TR mice but not in the corresponding APOE3 or APOE-deficient (KO) mice, APOER2 levels in hippocampus are also reduced (Gilat-Frenkel et al., 2014). Regulating expression levels of APOER2 could therefore be considered as a good anti-AD strategy.

### ApoE Gene Therapy

Adeno-associated viruses (AAVs) can mediate gene transfer directly to the CNS (Ittner et al., 2019). AAVs have become the most widely used gene therapy vectors for the CNS due of their safety, nonpathogenic nature, and capability to infect dividing and quiescent cells *in vivo*, particularly neurons (Ittner et al., 2019). An ongoing trial is scheduled to start soon to test the safety of AAV-APOE2 expression in APOE4 carriers^1^. Estimated completion date of this trial is December 2021. Patients will be injected with AAV-APOE2 in the cisterna magna and then followed for at least 2 years about their general health. This trial was made possible because previous studies in animals including APP/PS1 and Tg2576 mice showed that AAV-APOE2 reduces Aβ load after intracerebral administration of AAV-APOE4 (Hudry et al., 2013). Another group performed an intracerebral injection of AAV-APOE2 to APP/PS1/APOE4 TR mouse and reported that it reduced Aβ deposition (Zhao L. et al., 2016). More recently, a group used a non-viral delivery of plasmid encoding apoE2 (pApoE2) in the brain of mice using liposomes and showed a significant increase of apoE levels in the brain of mice with one single injection (dos Santos Rodrigues et al., 2019). Together these studies show that increasing the expression of apoE2, but not apoE4, could be efficient in reducing Aβ pathology.

While apoE-targeted therapies remain in an early phase of development, they hold great promises in the fight against LOAD. Up-regulation of apoE3 is likely to support synapses and other apoE-related functions, while down-regulation of apoE4 decreases its toxic effects and minimize Aβ deposition. The isoform-specific targeting approach would be an encouraging strategy for treating AD due to the differential functions of apoE isoforms in AD pathogenesis (Zhao et al., 2018a).

### Current Challenges and Considerations in Light of apoE-Targeted Therapies

All the recent phase three clinical trials for AD failed, highlighting that this challenge always remains a priority. It is likely that AD has a spectrum of diseases without a common trigger, with slightly different initiators, accelerators and exacerbators (Suidan and Ramaswamy, 2019). The mystery and ambiguity around the cause of AD is primarily what to blame for the lack of successful current therapeutics. It is highly probably that a cocktail of drugs for different targets might be required and adapted along with the disease progression.

One limitation in the recent studies is the small sample size with regards to the APOE genotype stratification. Two important questions in the field remain to be addressed: Is there a specific patient population for which an apoE-directed therapy would benefit the most? At what stage of the disease this therapy could be most successful? People with one copy of APOE2 have half the risk of developing LOAD as opposed to those with the most common ε3/ε3 genotype. We do not know whether the risk of ε2/ε2 is substantially lower than that of ε2/ε3 but new studies specifically focused on APOE2 are likely to reinvigorate interest among drug manufacturers. While it may be advantageous to increase apoE2 levels in the brain, long-term expression of APOE2 could increase the risk of cerebral amyloid angiopathy (CAA), CAA-associated intracerebral hemorrhagic and possibly primary tauopathy (Yamazaki et al., 2019).

Another limitation is the lack of understanding concerning the relationship between apoE lipidation status and markers of LOAD progression, such as Aβ ratio, phosphorylated tau, total tau protein, and cognitive end points. ApoE biology is highly complex and several factors must be addressed when targeting this protein in LOAD, in particular the lipidation status of apoE. It has proven difficult to develop molecules that modify the conformation of APOE4 to APOE3 or APOE2 because the variable degree of lipidation of APOE may affect its tertiary conformation. On the other hand, for APOE4 homozygote patients, approaches aiming at apoE4 reduction may be sufficient (Suidan and Ramaswamy, 2019). It is possible that such therapies slow the rate of cognitive decline in APOE4 carriers, but it is likely that the neurodegenerative process will not be completely halted (Safieh et al., 2019). Modulating the quantity of apoE or peripheral expression of apoE receptors may also increase the risk of atherosclerosis, hyperlipidaemia, and cardiovascular problems due to defective lipoprotein metabolism.

While we strive to better understand LOAD and find successful therapeutics, maintaining a healthy lifestyle (nutritional guidance, physical exercise, cognitive training, and management of metabolic and vascular risk factors) improve memory and cognitive function of older people carrying APOE4 (Solomon et al., 2018) and can assist with the onset of disease and symptoms. Indeed, not all APOE4 carriers will develop AD hence supporting that there are potential lifestyle conditions lowering the expression of the disease in this population.

## Conclusion

Carrying APOE4 is the major genetic risk factor for developing LOAD, although not everyone carrying APOE4 develops the disease. APOE not only impacts lipid metabolism but various CNS functions in an isoform-dependent manner. In addition of controlling blood cholesterol levels, apoE proteins also regulate Aβ deposition, aggregation and clearance. However, the exact molecular mechanisms behind Aβ regulation observed in human and animal models remain to be elucidated. It is still unclear whether APOE4 allele affects LOAD pathogenesis by a gain of toxic functions or a loss of defensive functions (or a combination of both). To date, no drugs have been developed to cure/delay AD or to target apoE4 pathways, and a long list of failures already pave the road to the discovery of successful LOAD therapies. This multifactorial disease might require a multi-target treatment likely to be adapted toward the disease progression. The current apoE-targeted strategies need to consider apoE lipidation and global lipid homeostasis in the periphery and into the brain. Combined therapy of increased lipidation with simultaneously decreasing lipid-free apoE4 would be an appealing approach to prevent the progression of AD. Exploring the biology of apoE isoforms may also provide more promising approaches. Finally, improving the lifestyle and diet also need to be considered to minimize the risks associated with the APOE4 isoform. Therefore, there is a need to generate fundamental knowledge not specifically oriented on one biomarker such as Aβ but to adopt an integrative systematic approach to tackle the understanding of this complex disease.

## Author Contributions

MAH, MP, and BL designed the review. MAH prepared the first draft. BL and MP revised and improved the draft. All authors read and approved the final submitted version of the manuscript.

## Conflict of Interest

The authors declare that the research was conducted in the absence of any commercial or financial relationships that could be construed as a potential conflict of interest.

## Footnotes

1 https://clinicaltrials.gov/ct2/show/NCT03634007

## References

[B1] Achariyar T. M. Li B. Peng W. Verghese P. B. Shi Y. McConnell E. (2016). Glymphatic distribution of CSF-derived apoE into brain is isoform specific and suppressed during sleep deprivation. *Mol. Neurodegener.* 11 1–20. 10.1186/s13024-016-0138-8 27931262 PMC5146863

[B2] Aizenstein H. J. Nebes R. D. Saxton J. A. Price J. C. Mathis C. A. Tsopelas N. D. (2008). Frequent amyloid deposition without significant cognitive impairment among the elderly. *Arch. Neurol.* 65 1509–1517. 10.1001/archneur.65.11.1509 19001171 PMC2636844

[B3] Altmann A. Tian L. Henderson V. W. Greicius M. D. Investigators A. D. N. I. (2014). Sex modifies the APOE-related risk of developing Alzheimer disease. *Ann. Neurol.* 75 563–573. 10.1002/ana.24135 24623176 PMC4117990

[B4] Ancelin M.-L. Ripoche E. Dupuy A.-M. Samieri C. Rouaud O. Berr C. (2014). Gender-specific associations between lipids and cognitive decline in the elderly. *Eur. Neuropsychopharmacol.* 24 1056–1066. 10.1016/j.euroneuro.2014.02.003 24721317

[B5] Arenaza-Urquijo E. M. Przybelski S. A. Lesnick T. L. Graff-Radford J. Machulda M. M. Knopman D. S. (2019). The metabolic brain signature of cognitive resilience in the 80+: beyond Alzheimer pathologies. *Brain* 142 1134–1147. 10.1093/brain/awz037 30851100 PMC6439329

[B6] Baker-Nigh A. T. Mawuenyega K. G. Bollinger J. G. Ovod V. Kasten T. Franklin E. E. (2016). Human central nervous system (CNS) ApoE isoforms are increased by age, differentially altered by amyloidosis, and relative amounts reversed in the CNS compared with plasma. *J. Biol. Chem.* 291 27204–27218. 10.1074/jbc.m116.721779 27793990 PMC5207148

[B7] Barnes L. L. Wilson R. S. Bienias J. L. Schneider J. A. Evans D. A. Bennett D. A. (2005). Sex differences in the clinical manifestations of Alzheimer disease pathology. *Arch. Gen. Psychiatry* 62 685–691. 10.1001/archpsyc.62.6.685 15939846

[B8] Belloy M. E. Napolioni V. Greicius M. D. (2019). A quarter century of APOE and Alzheimer’s disease: progress to date and the path forward. *Neuron* 101 820–838. 10.1016/j.neuron.2019.01.056 30844401 PMC6407643

[B9] Boehm-Cagan A. Bar R. Liraz O. Bielicki J. K. Johansson J. O. Michaelson D. M. (2016). ABCA1 agonist reverses the ApoE4-driven cognitive and brain pathologies. *J. Alzheimers Dis.* 54 1219–1233. 10.3233/jad-160467 27567858

[B10] Boisvert M. M. Erikson G. A. Shokhirev M. N. Allen N. J. (2018). The aging astrocyte transcriptome from multiple regions of the mouse brain. *Cell Rep.* 22 269–285. 10.1016/j.celrep.2017.12.039 29298427 PMC5783200

[B11] Brodbeck J. McGuire J. Liu Z. Meyer-Franke A. Balestra M. E. Jeong D. (2011). Structure-dependent impairment of intracellular apolipoprotein E4 trafficking and its detrimental effects are rescued by small-molecule structure correctors. *J. Biol. Chem.* 286 17217–17226. 10.1074/jbc.m110.217380 21454574 PMC3089564

[B12] Bu G. (2009). Apolipoprotein E and its receptors in Alzheimer’s disease: pathways, pathogenesis and therapy. *Nat. Rev. Neurosci.* 10 333–344. 10.1038/nrn2620 19339974 PMC2908393

[B13] Cam J. A. Bu G. (2006). Modulation of β-amyloid precursor protein trafficking and processing by the low density lipoprotein receptor family. *Mol. Neurodegener.* 1 1–13. 10.1186/1750-1326-1-8 16930455 PMC1563464

[B14] Cam J. A. Zerbinatti C. V. Knisely J. M. Hecimovic S. Li Y. Bu G. (2004). The low density lipoprotein receptor-related protein 1B retains β-amyloid precursor protein at the cell surface and reduces amyloid-β peptide production. *J. Biol. Chem.* 279 29639–29646. 10.1074/jbc.m313893200 15126508

[B15] Cao J. El Gaamouch F. Meabon J. S. Meeker K. D. Zhu L. Zhong M. B. (2017). ApoE4-associated phospholipid dysregulation contributes to development of Tau hyper-phosphorylation after traumatic brain injury. *Sci. Rep.* 7 1–12. 10.1038/s41598-017-11654-7 28900205 PMC5595858

[B16] Castellano J. M. Kim J. Stewart F. R. Jiang H. DeMattos R. B. Patterson B. W. (2011). Human apoE isoforms differentially regulate brain amyloid-β peptide clearance. *Sci. Transl. Med.* 3:89ra57. 10.1126/scitranslmed.3002156 21715678 PMC3192364

[B17] Cesa L. C. Mapp A. K. Gestwicki J. E. (2015). Direct and propagated effects of small molecules on protein–protein interaction networks. *Front. Bioeng. Biotechnol.* 3:119. 10.3389/fbioe.2015.00119 26380257 PMC4547496

[B18] Chen Y. Durakoglugil M. S. Xian X. Herz J. (2010). ApoE4 reduces glutamate receptor function and synaptic plasticity by selectively impairing ApoE receptor recycling. *Proc. Natl. Acad. Sci. U.S.A.* 107 12011–12016. 10.1073/pnas.0914984107 20547867 PMC2900641

[B19] Chernick D. Ortiz-Valle S. Jeong A. Qu W. Li L. (2019). Peripheral versus central nervous system APOE in Alzheimer’s disease: interplay across the blood-brain barrier. *Neurosci. Lett.* 708:134306. 10.1016/j.neulet.2019.134306 31181302 PMC6693948

[B20] Chernick D. Ortiz-Valle S. Jeong A. Swaminathan S. K. Kandimalla K. K. Rebeck G. W. (2018). High-density lipoprotein mimetic peptide 4F mitigates amyloid-β-induced inhibition of apolipoprotein E secretion and lipidation in primary astrocytes and microglia. *J. Neurochem.* 147 647–662. 10.1111/jnc.14554 30028014 PMC6314045

[B21] Chew H. Solomon V. A. Fonteh A. N. (2020). Involvement of lipids in Alzheimer’s disease pathology and potential therapies. *Front. Physiol.* 11:598. 10.3389/fphys.2020.00598 32581851 PMC7296164

[B22] Chouinard-Watkins R. Rioux-Perreault C. Fortier M. Tremblay-Mercier J. Zhang Y. Lawrence P. (2013). Disturbance in uniformly 13 C-labelled DHA metabolism in elderly human subjects carrying the apoE ε4 allele. *Br. J. Nutr.* 110 1751–1759. 10.1017/s0007114513001268 23631810

[B23] Christensen E. I. Gliemann J. Moestrup S. K. (1992). Renal tubule gp330 is a calcium binding receptor for endocytic uptake of protein. *J. Histochem. Cytochem.* 40 1481–1490. 10.1177/40.10.1382088 1382088

[B24] Corbo R. M. Scacchi R. (1999). Apolipoprotein E (APOE) allele distribution in the world. Is APOE^∗^ 4 a ‘thrifty’allele? *Ann. Hum. Genet.* 63 301–310. 10.1046/j.1469-1809.1999.6340301.x 10738542

[B25] Corder E. H. Ghebremedhin E. Taylor M. G. Thal D. R. Ohm T. G. Braak H. (2004). The biphasic relationship between regional brain senile plaque and neurofibrillary tangle distributions: modification by age, sex, and APOE polymorphism. *Ann. N. Y. Acad. Sci.* 1019 24–28. 10.1196/annals.1297.005 15246987

[B26] Cruchaga C. Kauwe J. S. Nowotny P. Bales K. Pickering E. H. Mayo K. (2012). Cerebrospinal fluid APOE levels: an endophenotype for genetic studies for Alzheimer’s disease. *Hum. Mol. Genet.* 21 4558–4571. 10.1093/hmg/dds296 22821396 PMC3459471

[B27] Cui W. Sun Y. Wang Z. Xu C. Xu L. Wang F. (2011). Activation of liver X receptor decreases BACE1 expression and activity by reducing membrane cholesterol levels. *Neurochem. Res.* 36 1910–1921. 10.1007/s11064-011-0513-3 21630010

[B28] DeVos S. L. Miller R. L. Schoch K. M. Holmes B. B. Kebodeaux C. S. Wegener A. J. (2017). Tau reduction prevents neuronal loss and reverses pathological tau deposition and seeding in mice with tauopathy. *Sci. Transl. Med.* 9:eaag0481. 10.1126/scitranslmed.aag0481 28123067 PMC5792300

[B29] Dlugosz P. Nimpf J. (2018). The reelin receptors apolipoprotein E receptor 2 (ApoER2) and VLDL receptor. *Int. J. Mol. Sci.* 19:3090. 10.3390/ijms19103090 30304853 PMC6213145

[B30] dos Santos Rodrigues B. Kanekiyo T. Singh J. (2019). ApoE-2 brain-targeted gene therapy through transferrin and penetratin tagged liposomal nanoparticles. *Pharm. Res.* 36:161. 10.1007/s11095-019-2691-7 31529284 PMC10150442

[B31] Egert S. Rimbach G. Huebbe P. (2012). ApoE genotype: from geographic distribution to function and responsiveness to dietary factors. *Proc. Nutr. Soc.* 71 410–424. 10.1017/s0029665112000249 22564824

[B32] Etique N. Verzeaux L. Dedieu S. Emonard H. (2013). LRP-1: a checkpoint for the extracellular matrix proteolysis. *BioMed Res. Int.* 2013:152163. 10.1155/2013/152163 23936774 PMC3723059

[B33] Farrer L. A. Cupples L. A. Haines J. L. Hyman B. Kukull W. A. Mayeux R. (1997). Effects of age, sex, and ethnicity on the association between apolipoprotein E genotype and Alzheimer disease: a meta-analysis. *Jama* 278 1349–1356. 10.1001/jama.1997.03550160069041 9343467

[B34] Fernandez C. G. Hamby M. E. McReynolds M. L. Ray W. J. (2019). The role of APOE4 in disrupting the homeostatic functions of astrocytes and microglia in aging and Alzheimer’s disease. *Front. Aging Neurosci.* 11:14. 10.3389/fnagi.2019.00014 30804776 PMC6378415

[B35] Flowers S. A. Rebeck G. W. (2020). APOE in the normal brain. *Neurobiol. Dis.* 136:104724. 10.1016/j.nbd.2019.104724 31911114 PMC7002287

[B36] Frieden C. Garai K. (2012). Structural differences between apoE3 and apoE4 may be useful in developing therapeutic agents for Alzheimer’s disease. *Proc. Natl. Acad. Sci. U.S.A.* 109 8913–8918. 10.1073/pnas.1207022109 22615372 PMC3384159

[B37] Gilat-Frenkel M. Boehm-Cagan A. Liraz O. Xian X. Herz J. Michaelson D. M. (2014). Involvement of the Apoer2 and Lrp1 receptors in mediating the pathological effects of ApoE4 in vivo. *Curr. Alzheimer Res.* 11 549–557. 10.2174/1567205010666131119232444 24251389 PMC4065855

[B38] Goldstein J. L. Brown M. S. (2015). A century of cholesterol and coronaries: from plaques to genes to statins. *Cell* 161 161–172. 10.1016/j.cell.2015.01.036 25815993 PMC4525717

[B39] Gottschalk W. K. Mihovilovic M. Roses A. D. Chiba-Falek O. (2016). The role of upregulated APOE in Alzheimer’s disease etiology. *J. Alzheimers Dis. Park.* 6:209. 10.4172/2161-0460.1000209 27104063 PMC4836841

[B40] Guptill J. T. Raja S. M. Boakye-Agyeman F. Noveck R. Ramey S. Tu T. M. (2017). Phase 1 randomized, double-blind, placebo-controlled study to determine the safety, tolerability, and pharmacokinetics of a single escalating dose and repeated doses of CN-105 in healthy adult subjects. *J. Clin. Pharmacol.* 57 770–776. 10.1002/jcph.853 27990643 PMC5524215

[B41] Hara M. Matsushima T. Satoh H. Iso-o N. Noto H. Togo M. (2003). Isoform-dependent cholesterol efflux from macrophages by apolipoprotein E is modulated by cell surface proteoglycans. *Arterioscler. Thromb. Vasc. Biol.* 23 269–274. 10.1161/01.atv.0000054199.78458.4b 12588770

[B42] Hatters D. M. Peters-Libeu C. A. Weisgraber K. H. (2006a). Apolipoprotein E structure: insights into function. *Trends Biochem. Sci.* 31 445–454. 10.1016/j.tibs.2006.06.008 16820298

[B43] Hatters D. M. Zhong N. Rutenber E. Weisgraber K. H. (2006b). Amino-terminal domain stability mediates apolipoprotein E aggregation into neurotoxic fibrils. *J. Mol. Biol.* 361 932–944. 10.1016/j.jmb.2006.06.080 16890957

[B44] Hauser P. S. Narayanaswami V. Ryan R. O. (2011). Apolipoprotein E: from lipid transport to neurobiology. *Prog. Lipid Res.* 50 62–74. 10.1016/j.plipres.2010.09.001 20854843 PMC3022415

[B45] He X. Cooley K. Chung C. H. Dashti N. Tang J. (2007). Apolipoprotein receptor 2 and X11α/β mediate apolipoprotein E-induced endocytosis of amyloid-β precursor protein and β-secretase, leading to amyloid-β production. *J. Neurosci.* 27 4052–4060. 10.1523/jneurosci.3993-06.2007 17428983 PMC6672528

[B46] Heinsinger N. M. Gachechiladze M. A. Rebeck G. W. (2016). Apolipoprotein E genotype affects size of ApoE complexes in cerebrospinal fluid. *J. Neuropathol. Exp. Neurol.* 75 918–924. 10.1093/jnen/nlw067 27516118 PMC6996536

[B47] Hinrich A. J. Jodelka F. M. Chang J. L. Brutman D. Bruno A. M. Briggs C. A. (2016). Therapeutic correction of ApoER2 splicing in Alzheimer’s disease mice using antisense oligonucleotides. *EMBO Mol. Med.* 8 328–345. 10.15252/emmm.201505846 26902204 PMC4818756

[B48] Hoe H.-S. Freeman J. Rebeck G. W. (2006). Apolipoprotein E decreases tau kinases and phospho-tau levels in primary neurons. *Mol. Neurodegener.* 1:18. 10.1186/1750-1326-1-18 17166269 PMC1713232

[B49] Holtzman D. M. Herz J. Bu G. (2012). Apolipoprotein E and apolipoprotein E receptors: normal biology and roles in Alzheimer disease. *Cold Spring Harb. Perspect. Med.* 2:a006312. 10.1101/cshperspect.a006312 22393530 PMC3282491

[B50] Hsu M. Dedhia M. Crusio W. E. Delprato A. (2019). Sex differences in gene expression patterns associated with the APOE4 allele. *F1000Research* 8:387. 10.12688/f1000research.18671.2 31448102 PMC6685458

[B51] Hu J. Liu C.-C. Chen X.-F. Zhang Y. Xu H. Bu G. (2015). Opposing effects of viral mediated brain expression of apolipoprotein E2 (apoE2) and apoE4 on apoE lipidation and Aβ metabolism in apoE4-targeted replacement mice. *Mol. Neurodegener.* 10 1–11. 10.1016/j.bbr.2004.09.019 25871773 PMC4356137

[B52] Huang Y.-W. A. Zhou B. Wernig M. Südhof T. C. (2017). ApoE2, ApoE3, and ApoE4 differentially stimulate APP transcription and Aβ secretion. *Cell* 168 427–441. 10.1016/j.cell.2016.12.044 28111074 PMC5310835

[B53] Huang Z. H. Reardon C. A. Getz G. S. Maeda N. Mazzone T. (2015). Selective suppression of adipose tissue apoE expression impacts systemic metabolic phenotype and adipose tissue inflammation. *J. Lipid Res.* 56 215–226. 10.1194/jlr.m050567 25421060 PMC4306677

[B54] Hubin E. Verghese P. B. van Nuland N. Broersen K. (2019). Apolipoprotein E associated with reconstituted high-density lipoprotein-like particles is protected from aggregation. *FEBS Lett.* 593 1144–1153. 10.1002/1873-3468.13428 31058310 PMC6617784

[B55] Hudry E. Dashkoff J. Roe A. D. Takeda S. Koffie R. M. Hashimoto T. (2013). Gene transfer of human Apoe isoforms results in differential modulation of amyloid deposition and neurotoxicity in mouse brain. *Sci. Transl. Med.* 5:212ra161. 10.1126/scitranslmed.3007000 24259049 PMC4334150

[B56] Huynh T.-P. V. Liao F. Francis C. M. Robinson G. O. Serrano J. R. Jiang H. (2017). Age-dependent effects of apoE reduction using antisense oligonucleotides in a model of β-amyloidosis. *Neuron* 96 1013–1023. 10.1016/j.neuron.2017.11.014 29216448 PMC5728673

[B57] Ittner L. M. Klugmann M. Ke Y. D. (2019). Adeno-associated virus-based Alzheimer’s disease mouse models and potential new therapeutic avenues. *Br. J. Pharmacol.* 176 3649–3665. 10.1111/bph.14637 30817847 PMC6715621

[B58] Kanekiyo T. Cirrito J. R. Liu C.-C. Shinohara M. Li J. Schuler D. R. (2013). Neuronal clearance of amyloid-β by endocytic receptor LRP1. *J. Neurosci.* 33 19276–19283. 10.1523/jneurosci.3487-13.2013 24305823 PMC3850043

[B59] Kanekiyo T. Liu C.-C. Shinohara M. Li J. Bu G. (2012). LRP1 in brain vascular smooth muscle cells mediates local clearance of Alzheimer’s amyloid-β. *J. Neurosci.* 32 16458–16465. 10.1523/jneurosci.3987-12.2012 23152628 PMC3508699

[B60] Kanekiyo T. Xu H. Bu G. (2014). ApoE and Aβ in Alzheimer’s disease: accidental encounters or partners? *Neuron* 81 740–754. 10.1016/j.neuron.2014.01.045 24559670 PMC3983361

[B61] Kang S. S. Ebbert M. T. Baker K. E. Cook C. Wang X. Sens J. P. (2018). Microglial translational profiling reveals a convergent APOE pathway from aging, amyloid, and tau. *J. Exp. Med.* 215 2235–2245. 10.1084/jem.20180653 30082275 PMC6122978

[B62] Kim J. Basak J. M. Holtzman D. M. (2009). The Role of Apolipoprotein E in Alzheimer’s Disease. *Neuron* 63 287–303. 10.1016/j.neuron.2009.06.026 19679070 PMC3044446

[B63] Kim J. Eltorai A. E. Jiang H. Liao F. Verghese P. B. Kim J. (2012). Anti-apoE immunotherapy inhibits amyloid accumulation in a transgenic mouse model of Aβ amyloidosis. *J. Exp. Med.* 209 2149–2156. 10.1084/jem.20121274 23129750 PMC3501350

[B64] Kloske C. M. Wilcock D. M. (2020). The important interface between apolipoprotein e and neuroinflammation in Alzheimer’s disease. *Front. Immunol.* 11:754. 10.3389/fimmu.2020.00754 PMC7203730 32425941

[B65] Koch S. Donarski N. Goetze K. Kreckel M. Stuerenburg H.-J. Buhmann C. (2001). Characterization of four lipoprotein classes in human cerebrospinal fluid. *J. Lipid Res.* 42 1143–1151. 10.1016/s0022-2275(20)31605-9 11441143

[B66] Kockx M. Jessup W. Kritharides L. (2008). Regulation of endogenous apolipoprotein E secretion by macrophages. *Arterioscler. Thromb. Vasc. Biol.* 28 1060–1067. 10.1161/atvbaha.108.164350 18388328

[B67] Kounnas M. Z. Haudenschild C. C. Strickland D. K. Argraves W. S. (1994). Immunological localization of glycoprotein 330, low density lipoprotein receptor related protein and 39 kDa receptor associated protein in embryonic mouse tissues. *Vivo Athens Greece* 8 343–351. 7803716

[B68] Krishnamurthy K. Cantillana V. Wang H. Sullivan P. M. Kolls B. J. Ge X. (2020). ApoE mimetic improves pathology and memory in a model of Alzheimer’s disease. *Brain Res.* 1733:146685. 10.1016/j.brainres.2020.146685 32007397

[B69] Lane-Donovan C. Herz J. (2017). The ApoE receptors Vldlr and Apoer2 in central nervous system function and disease. *J. Lipid Res.* 58 1036–1043. 10.1194/jlr.r075507 28292942 PMC5454520

[B70] Laskowitz D. T. Wang H. Chen T. Lubkin D. T. Cantillana V. Tu T. M. (2017). Neuroprotective pentapeptide CN-105 is associated with reduced sterile inflammation and improved functional outcomes in a traumatic brain injury murine model. *Sci. Rep.* 7:46461. 10.1038/srep46461 28429734 PMC5399447

[B71] Liao F. Hori Y. Hudry E. Bauer A. Q. Jiang H. Mahan T. E. (2014). Anti-ApoE antibody given after plaque onset decreases Aβ accumulation and improves brain function in a mouse model of Aβ amyloidosis. *J. Neurosci.* 34 7281–7292. 10.1523/jneurosci.0646-14.2014 24849360 PMC4028501

[B72] Liao F. Li A. Xiong M. Bien-Ly N. Jiang H. Zhang Y. (2018). Targeting of nonlipidated, aggregated apoE with antibodies inhibits amyloid accumulation. *J. Clin. Invest.* 128 2144–2155. 10.1172/jci96429 29600961 PMC5919821

[B73] Lim W. L. F. Huynh K. Chatterjee P. Martins I. Jayawardana K. S. Giles C. (2020). Relationships between plasma lipids species, gender, risk factors, and Alzheimer’s disease. *J. Alzheimers Dis.* 76 303–315. 10.3233/jad-191304 32474467 PMC7369125

[B74] Lin Y.-T. Seo J. Gao F. Feldman H. M. Wen H.-L. Penney J. (2018). APOE4 causes widespread molecular and cellular alterations associated with Alzheimer’s disease phenotypes in human iPSC-derived brain cell types. *Neuron* 98 1141–1154. 10.1016/j.neuron.2018.05.008 29861287 PMC6023751

[B75] Liu C.-C. Hu J. Zhao N. Wang J. Wang N. Cirrito J. R. (2017). Astrocytic LRP1 mediates brain Aβ clearance and impacts amyloid deposition. *J. Neurosci.* 37 4023–4031. 10.1523/jneurosci.3442-16.2017 28275161 PMC5391682

[B76] Liu C.-C. Kanekiyo T. Xu H. Bu G. (2013). Apolipoprotein E and Alzheimer disease: risk, mechanisms and therapy. *Nat. Rev. Neurol.* 9 106–118. 10.1038/nrneurol.2012.263 23296339 PMC3726719

[B77] Liu J. Zhou G. Kolls B. J. Tan Y. Fang C. Wang H. (2018). Apolipoprotein E mimetic peptide CN-105 improves outcome in a murine model of SAH. *Stroke Vasc. Neurol.* 3 222–230. 10.1136/svn-2018-000152 30637128 PMC6312076

[B78] Liu S. Breitbart A. Sun Y. Mehta P. D. Boutajangout A. Scholtzova H. (2014). Blocking the apolipoprotein E/amyloid β interaction in triple transgenic mice ameliorates Alzheimer’s disease related amyloid β and tau pathology. *J. Neurochem.* 128 577–591. 10.1111/jnc.12484 24117759 PMC3946231

[B79] Lohner S. Fekete K. Marosvölgyi T. Decsi T. (2013). Gender differences in the long-chain polyunsaturated fatty acid status: systematic review of 51 publications. *Ann. Nutr. Metab.* 62 98–112. 10.1159/000345599 23327902

[B80] Ma Q. Zhao Z. Sagare A. P. Wu Y. Wang M. Owens N. C. (2018). Blood-brain barrier-associated pericytes internalize and clear aggregated amyloid-β42 by LRP1-dependent apolipoprotein E isoform-specific mechanism. *Mol. Neurodegener.* 13 1–13. 10.1186/s13024-018-0286-0 30340601 PMC6194676

[B81] Mahley R. W. Rall S. C. Jr. (2000). Apolipoprotein E: far more than a lipid transport protein. *Annu. Rev. Genomics Hum. Genet.* 1 507–537. 10.1146/annurev.genom.1.1.507 11701639

[B82] Marottoli F. M. Katsumata Y. Koster K. P. Thomas R. Fardo D. W. Tai L. M. (2017). Peripheral inflammation, apolipoprotein E4, and amyloid-β interact to induce cognitive and cerebrovascular dysfunction. *ASN Neuro* 9:1759091417719201 10.1177/1759091417719201 28707482 PMC5521356

[B83] Marschang P. Brich J. Weeber E. J. Sweatt J. D. Shelton J. M. Richardson J. A. (2004). Normal development and fertility of knockout mice lacking the tumor suppressor gene LRP1b suggest functional compensation by LRP1. *Mol. Cell. Biol.* 24 3782–3793. 10.1128/mcb.24.9.3782-3793.2004 15082773 PMC387731

[B84] Martínez-Morillo E. Hansson O. Atagi Y. Bu G. Minthon L. Diamandis E. P. (2014). Total apolipoprotein E levels and specific isoform composition in cerebrospinal fluid and plasma from Alzheimer’s disease patients and controls. *Acta Neuropathol. (Berl.)* 127 633–643. 10.1007/s00401-014-1266-2 24633805

[B85] McIntosh A. M. Bennett C. Dickson D. Anestis S. F. Watts D. P. Webster T. H. (2012). The apolipoprotein E (APOE) gene appears functionally monomorphic in chimpanzees (Pan troglodytes). *PLoS One* 7:47760. 10.1371/journal.pone.0047760 23112842 PMC3480407

[B86] Mesa-Herrera F. Taoro-González L. Valdés-Baizabal C. Diaz M. Marín R. (2019). Lipid and lipid raft alteration in aging and neurodegenerative diseases: a window for the development of new biomarkers. *Int. J. Mol. Sci.* 20:3810. 10.3390/ijms20153810 31382686 PMC6696273

[B87] Nelson T. J. Sen A. (2019). Apolipoprotein E particle size is increased in Alzheimer’s disease. *Alzheimers Dement. Diagn. Assess. Dis. Monit.* 11 10–18. 10.1016/j.dadm.2018.10.005 30581971 PMC6293020

[B88] Neu S. C. Pa J. Kukull W. Beekly D. Kuzma A. Gangadharan P. (2017). Apolipoprotein E genotype and sex risk factors for Alzheimer disease: a meta-analysis. *JAMA Neurol.* 74 1178–1189. 10.1001/jamaneurol.2017.2188 28846757 PMC5759346

[B89] Nguyen D. Dhanasekaran P. Nickel M. Nakatani R. Saito H. Phillips M. C. (2010). Molecular basis for the differences in lipid and lipoprotein binding properties of human apolipoproteins E3 and E4. *Biochemistry* 49 10881–10889. 10.1021/bi1017655 21114327 PMC3025481

[B90] Nickerson D. A. Taylor S. L. Fullerton S. M. Weiss K. M. Clark A. G. Stengård J. H. (2000). Sequence diversity and large-scale typing of SNPs in the human apolipoprotein E gene. *Genome Res.* 10 1532–1545. 10.1101/gr.146900 11042151 PMC310963

[B91] Osei-Hwedieh D. O. Amar M. Sviridov D. Remaley A. T. (2011). Apolipoprotein mimetic peptides: mechanisms of action as anti-atherogenic agents. *Pharmacol. Ther.* 130 83–91. 10.1016/j.pharmthera.2010.12.003 21172387 PMC3043134

[B92] Palmisano B. T. Zhu L. Eckel R. H. Stafford J. M. (2018). Sex differences in lipid and lipoprotein metabolism. *Mol. Metab.* 15 45–55. 10.1016/j.molmet.2018.05.008 29858147 PMC6066747

[B93] Pankiewicz J. E. Guridi M. Kim J. Asuni A. A. Sanchez S. Sullivan P. M. (2014). Blocking the apoE/Aβ interaction ameliorates Aβ-related pathology in APOE ε2 and ε4 targeted replacement Alzheimer model mice. *Acta Neuropathol. Commun.* 2:75. 10.1186/s40478-014-0075-0 24972680 PMC4174325

[B94] Payami H. Montee K. R. Kaye J. A. Bird T. D. Yu C.-E. Wijsman E. M. (1994). Alzheimer’s disease, apolipoprotein E4, and gender. *Jama* 271 1316–1317. 10.1001/jama.271.17.1316 8158809

[B95] Peng D. Song C. Reardon C. A. Liao S. Getz G. S. (2003). Lipoproteins produced by ApoE–/–astrocytes infected with adenovirus expressing human ApoE. *J. Neurochem.* 86 1391–1402. 10.1046/j.1471-4159.2003.01950.x 12950448

[B96] Phillips M. C. (2014). Apolipoprotein E isoforms and lipoprotein metabolism. *IUBMB Life* 66 616–623. 10.1002/iub.1314 25328986

[B97] Prasad H. Rao R. (2018). Amyloid clearance defect in ApoE4 astrocytes is reversed by epigenetic correction of endosomal pH. *Proc. Natl. Acad. Sci. U.S.A.* 115 E6640–E6649. 10.1073/pnas.1801612115 29946028 PMC6048470

[B98] Qian J. Wolters F. J. Beiser A. Haan M. Ikram M. A. Karlawish J. (2017). APOE-related risk of mild cognitive impairment and dementia for prevention trials: an analysis of four cohorts. *PLoS Med.* 14:e1002254. 10.1371/journal.pmed.1002254 28323826 PMC5360223

[B99] Qosa H. Abuznait A. H. Hill R. A. Kaddoumi A. (2012). Enhanced brain amyloid-β clearance by rifampicin and caffeine as a possible protective mechanism against Alzheimer’s disease. *J. Alzheimers Dis.* 31 151–165. 10.3233/jad-2012-120319 22504320 PMC3902015

[B100] Rapp A. Gmeiner B. Hüttinger M. (2006). Implication of apoE isoforms in cholesterol metabolism by primary rat hippocampal neurons and astrocytes. *Biochimie* 88 473–483. 10.1016/j.biochi.2005.10.007 16376010

[B101] Rawat V. Wang S. Sima J. Bar R. Liraz O. Gundimeda U. (2019). ApoE4 alters ABCA1 membrane trafficking in astrocytes. *J. Neurosci.* 39 9611–9622. 10.1523/jneurosci.1400-19.2019 31641056 PMC6880458

[B102] Rebeck G. W. (2017). The role of APOE on lipid homeostasis and inflammation in normal brains. *J. Lipid Res.* 58 1493–1499. 10.1194/jlr.r075408 28258087 PMC5538293

[B103] Reiman E. M. Arboleda-Velasquez J. F. Quiroz Y. T. Huentelman M. J. Beach T. G. Caselli R. J. (2020). Exceptionally low likelihood of Alzheimer’s dementia in APOE2 homozygotes from a 5,000-person neuropathological study. *Nat. Commun.* 11 1–11. 10.1038/s41467-019-14279-8 32015339 PMC6997393

[B104] Riddell D. R. Zhou H. Atchison K. Warwick H. K. Atkinson P. J. Jefferson J. (2008). Impact of apolipoprotein E (ApoE) polymorphism on brain ApoE levels. *J. Neurosci.* 28 11445–11453. 10.1523/jneurosci.1972-08.2008 18987181 PMC6671315

[B105] Ruiz J. Kouiavskaia D. Migliorini M. Robinson S. Saenko E. L. Gorlatova N. (2005). The apoE isoform binding properties of the VLDL receptor reveal marked differences from LRP and the LDL receptor. *J. Lipid Res.* 46 1721–1731. 10.1194/jlr.m500114-jlr200 15863833

[B106] Russell D. W. Halford R. W. Ramirez D. M. O. Shah R. Kotti T. (2009). Cholesterol 24-hydroxylase: an enzyme of cholesterol turnover in the brain. *Annu. Rev. Biochem.* 78 1017–1040. 10.1146/annurev.biochem.78.072407.103859 19489738 PMC2837268

[B107] Safieh M. Korczyn A. D. Michaelson D. M. (2019). ApoE4: an emerging therapeutic target for Alzheimer’s disease. *BMC Med.* 17:64. 10.1186/s12916-019-1299-4 30890171 PMC6425600

[B108] Sawmiller D. Habib A. Hou H. Mori T. Fan A. Tian J. (2019). A novel apolipoprotein E antagonist functionally blocks apolipoprotein E interaction with N-terminal amyloid precursor protein, reduces β-amyloid-associated pathology, and improves cognition. *Biol. Psychiatry* 86 208–220. 10.1016/j.biopsych.2019.04.026 31208706 PMC6642011

[B109] Schoch K. M. Miller T. M. (2017). Antisense oligonucleotides: translation from mouse models to human neurodegenerative diseases. *Neuron* 94 1056–1070. 10.1016/j.neuron.2017.04.010 28641106 PMC5821515

[B110] Shi Y. Yamada K. Liddelow S. A. Smith S. T. Zhao L. Luo W. (2017). ApoE4 markedly exacerbates tau-mediated neurodegeneration in a mouse model of tauopathy. *Nature* 549 523–527. 10.1038/nature24016 28959956 PMC5641217

[B111] Shinohara M. Sato N. Kurinami H. Takeuchi D. Takeda S. Shimamura M. (2010). Reduction of brain β-amyloid (Aβ) by fluvastatin, a hydroxymethylglutaryl-CoA reductase inhibitor, through increase in degradation of amyloid precursor protein C-terminal fragments (APP-CTFs) and Aβ clearance. *J. Biol. Chem.* 285 22091–22102. 10.1074/jbc.m110.102277 20472556 PMC2903370

[B112] Singh P. P. Singh M. Mastana S. S. (2006). APOE distribution in world populations with new data from India and the UK. *Ann. Hum. Biol.* 33 279–308. 10.1080/03014460600594513 17092867

[B113] Singh R. K. Haka A. S. Asmal A. Barbosa-Lorenzi V. C. Grosheva I. Chin H. F. (2020). TLR4 (toll-like receptor 4)-dependent signaling drives extracellular catabolism of LDL (low-density lipoprotein) aggregates. *Arterioscler. Thromb. Vasc. Biol.* 40 86–102. 10.1161/atvbaha.119.313200 31597445 PMC6928397

[B114] Solomon A. Turunen H. Ngandu T. Peltonen M. Levälahti E. Helisalmi S. (2018). Effect of the apolipoprotein E genotype on cognitive change during a multidomain lifestyle intervention: a subgroup analysis of a randomized clinical trial. *JAMA Neurol.* 75 462–470. 10.1001/jamaneurol.2017.4365 29356827 PMC5885273

[B115] Spuch C. Antequera D. Pascual C. Abilleira S. Blanco M. Moreno-Carretero M. J. (2015). Soluble megalin is reduced in cerebrospinal fluid samples of Alzheimer’s disease patients. *Front. Cell. Neurosci.* 9:134. 10.3389/fncel.2015.00134 25926771 PMC4397959

[B116] Suidan G. L. Ramaswamy G. (2019). Targeting apolipoprotein E for Alzheimer’s disease: an industry perspective. *Int. J. Mol. Sci.* 20:2161. 10.3390/ijms20092161 31052389 PMC6539182

[B117] Svennerholm L. Boström K. Jungbjer B. (1997). Changes in weight and compositions of major membrane components of human brain during the span of adult human life of Swedes. *Acta Neuropathol. (Berl.)* 94 345–352. 10.1007/s004010050717 9341935

[B118] Tachibana M. Holm M.-L. Liu C.-C. Shinohara M. Aikawa T. Oue H. (2019). APOE4-mediated amyloid-β pathology depends on its neuronal receptor LRP1. *J. Clin. Invest.* 129 1272–1277. 10.1172/jci124853 30741718 PMC6391135

[B119] Tachibana M. Shinohara M. Yamazaki Y. Liu C.-C. Rogers J. Bu G. (2016). Rescuing effects of RXR agonist bexarotene on aging-related synapse loss depend on neuronal LRP1. *Exp. Neurol.* 277 1–9. 10.1016/j.expneurol.2015.12.003 26688581 PMC4761336

[B120] Talwar P. Sinha J. Grover S. Agarwal R. Kushwaha S. Srivastava M. P. (2016). Meta-analysis of apolipoprotein E levels in the cerebrospinal fluid of patients with Alzheimer’s disease. *J. Neurol. Sci.* 360 179–187. 10.1016/j.jns.2015.12.004 26723997

[B121] Tarasoff-Conway J. M. Carare R. O. Osorio R. S. Glodzik L. Butler T. Fieremans E. (2015). Clearance systems in the brain—implications for Alzheimer disease. *Nat. Rev. Neurol.* 11:457. 10.1038/nrneurol.2015.119 26195256 PMC4694579

[B122] Tcw J. Liang S. A. Qian L. Pipalia N. H. Chao M. J. Bertelsen S. E. (2019). *Cholesterol and Matrisome Pathways Dysregulated in Human APOE ϵ4 Glia.* Rochester, NY: Social Science Research Network. 10.2139/ssrn.3435267

[B123] Teng Z. Guo Z. Zhong J. Cheng C. Huang Z. Wu Y. (2017). ApoE influences the blood-brain barrier through the NF-κB/MMP-9 pathway after traumatic brain injury. *Sci. Rep.* 7, 1–8. 10.1038/s41598-017-06932-3 28751738 PMC5532277

[B124] Theendakara V. Peters-Libeu C. A. Spilman P. Poksay K. S. Bredesen D. E. Rao R. V. (2016). Direct Transcriptional Effects of Apolipoprotein E. *J. Neurosci.* 36 685–700. 10.1523/JNEUROSCI.3562-15.2016 26791201 PMC4719010

[B125] Ulrich J. D. Burchett J. M. Restivo J. L. Schuler D. R. Verghese P. B. Mahan T. E. (2013). In vivo measurement of apolipoprotein E from the brain interstitial fluid using microdialysis. *Mol. Neurodegener.* 8 1–7. 10.1186/1750-1326-8-13 23601557 PMC3640999

[B126] van Veluw S. J. Shih A. Y. Smith E. E. Chen C. Schneider J. A. Wardlaw J. M. (2017). Detection, risk factors, and functional consequences of cerebral microinfarcts. *Lancet Neurol.* 16 730–740. 10.1016/s1474-4422(17)30196-5 28716371 PMC5861500

[B127] Vasilevskaya A. Taghdiri F. Burke C. Tarazi A. Naeimi S. A. Khodadadi M. (2020). Interaction of APOE4 alleles and PET tau imaging in former contact sport athletes. *NeuroImage Clin.* 26:102212. 10.1016/j.nicl.2020.102212 32097865 PMC7037542

[B128] Villeneuve S. Brisson D. Gaudet D. (2015). Influence of abdominal obesity on the lipid-lipoprotein profile in apoprotein E2/4 carriers: the effect of an apparent duality. *J. Lipids* 2015:742408. 10.1155/2015/742408 26605088 PMC4641183

[B129] Wang C. Najm R. Xu Q. Jeong D. Walker D. Balestra M. E. (2018). Gain of toxic apolipoprotein E4 effects in human iPSC-derived neurons is ameliorated by a small-molecule structure corrector. *Nat. Med.* 24 647–657. 10.1038/s41591-018-0004-z 29632371 PMC5948154

[B130] Williams H. C. Farmer B. C. Piron M. A. Walsh A. E. Bruntz R. C. Gentry M. S. (2020). APOE alters glucose flux through central carbon pathways in astrocytes. *Neurobiol. Dis.* 136:104742. 10.1016/j.nbd.2020.104742 31931141 PMC7044721

[B131] Wilson C. Wardell M. R. Weisgraber K. H. Mahley R. W. Agard D. A. (1991). Three-dimensional structure of the LDL receptor-binding domain of human apolipoprotein E. *Science* 252 1817–1822. 10.1126/science.2063194 2063194

[B132] Wolters F. J. Yang Q. Biggs M. L. Jakobsdottir J. Li S. Evans D. S. (2019). The impact of APOE genotype on survival: results of 38,537 participants from six population-based cohorts (E2-CHARGE). *PLoS One* 14:e0219668. 10.1371/journal.pone.0219668 31356640 PMC6663005

[B133] Wu L. Zhang X. Zhao L. (2018). Human apoe isoforms differentially modulate brain glucose and ketone body metabolism: Implications for Alzheimer’s disease risk reduction and early intervention. *J. Neurosci.* 38 6665–6681. 10.1523/JNEUROSCI.2262-17.2018 29967007 PMC6067075

[B134] Yajima R. Tokutake T. Koyama A. Kasuga K. Tezuka T. Nishizawa M. (2015). ApoE-isoform-dependent cellular uptake of amyloid-β is mediated by lipoprotein receptor LR11/SorLA. *Biochem. Biophys. Res. Commun.* 456 482–488. 10.1016/j.bbrc.2014.11.111 25482438

[B135] Yamazaki Y. Zhao N. Caulfield T. R. Liu C.-C. Bu G. (2019). Apolipoprotein E and Alzheimer disease: pathobiology and targeting strategies. *Nat. Rev. Neurol.* 15 501–518. 10.1038/s41582-019-0228-7 31367008 PMC7055192

[B136] Yang Y. G. Kim J. Y. Park S. J. Kim S. W. Jeon O. H. Kim D. S. (2007). Apolipoprotein E genotyping by multiplex tetra-primer amplification refractory mutation system PCR in single reaction tube. *J. Biotechnol.* 131 106–110. 10.1016/j.jbiotec.2007.06.001 17643539

[B137] Yao X. Vitek M. P. Remaley A. T. Levine S. J. (2012). Apolipoprotein mimetic peptides: a new approach for the treatment of asthma. *Front. Pharmacol.* 3:37. 10.3389/fphar.2012.00037 22408624 PMC3297834

[B138] Yassine H. N. Feng Q. Chiang J. Petrosspour L. M. Fonteh A. N. Chui H. C. (2016). ABCA1-mediated cholesterol efflux capacity to cerebrospinal fluid is reduced in patients with mild cognitive impairment and alzheimer’s disease. *J. Am. Heart Assoc.* 5:e002886. 10.1161/JAHA.115.002886 26873692 PMC4802440

[B139] Yassine H. N. Finch C. E. (2020). APOE alleles and diet in brain aging and Alzheimer’s disease. *Front. Aging Neurosci.* 12:150. 10.3389/fnagi.2020.00150 PMC7297981 32587511

[B140] Yu J.-T. Tan L. Hardy J. (2014). Apolipoprotein E in Alzheimer’s disease: an update. *Annu. Rev. Neurosci.* 37 79–100. 10.1093/jnen/59.9.751 24821312

[B141] Zhao J. Davis M. D. Martens Y. A. Shinohara M. Graff-Radford N. R. Younkin S. G. (2017). APOE ε4/ε4 diminishes neurotrophic function of human iPSC-derived astrocytes. *Hum. Mol. Genet.* 26 2690–2700. 10.1093/hmg/ddx155 28444230 PMC5886091

[B142] Zhao L. Gottesdiener A. J. Parmar M. Li M. Kaminsky S. M. Chiuchiolo M. J. (2016). Intracerebral adeno-associated virus gene delivery of apolipoprotein E2 markedly reduces brain amyloid pathology in Alzheimer’s disease mouse models. *Neurobiol. Aging* 44 159–172. 10.1016/j.neurobiolaging.2016.04.020 27318144

[B143] Zhao N. Liu C.-C. Qiao W. Bu G. (2018a). Apolipoprotein E, receptors, and modulation of Alzheimer’s disease. *Biol. Psychiatry* 83 347–357. 10.1016/j.biopsych.2017.03.003 28434655 PMC5599322

[B144] Zhao N. Liu C.-C. Van Ingelgom A. J. Linares C. Kurti A. Knight J. A. (2018b). APOE ε2 is associated with increased tau pathology in primary tauopathy. *Nat. Commun.* 9 1–11. 10.1038/s41467-018-06783-0 30348994 PMC6197187

[B145] Zhao N. Liu C.-C. Van Ingelgom A. J. Martens Y. A. Linares C. Knight J. A. (2017). Apolipoprotein E4 impairs neuronal insulin signaling by trapping insulin receptor in the endosomes. *Neuron* 96 115–129. 10.1016/j.neuron.2017.09.003 28957663 PMC5621659

